# The impact of patient navigation on glycemic control, adherence to self-care and knowledge about diabetes: an intervention study

**DOI:** 10.1186/s13098-023-01147-1

**Published:** 2023-08-17

**Authors:** Luciana Foppa, Betina Nemetz, Rosimeri de Matos, Josiane Schneiders, Gabriela Heiden Telo, Beatriz D. Schaan

**Affiliations:** 1 Hospital de Clínicas de Porto Alegre (HCPA), Rua Ramiro Barcelos 2350, Porto Alegre, RS 90035-003 Brazil; 2 Post-Graduate Program in Endocrinology, Universidade Federal do Rio Grande do Sul, Rua Ramiro Barcelos 2400, 2º Andar, Porto Alegre, RS 90035-003 Brazil; 3 Nurse School, Universidade Federal do Rio Grande do Sul, Rua Ramiro Barcelos, 2400, Porto Alegre, RS 90035-002 Brazil; 4 Hospital São Lucas da Pontifícia Universidade Católica do Rio Grande do Sul, Avenida Ipiranga, 6680, Jardim Botânico, Porto Alegre, RS 90619-900 Brazil; 5 Pontifícia Universidade Católica do Rio Grande do Sul, Avenida Ipiranga, 6681, Partenon, Porto Alegre, RS 90619-900, Brazil

**Keywords:** Patient navigation, Office nursing, Diabetes mellitus, type 1, Process assessment, health care, Health education

## Abstract

**Background:**

Patient navigation helps with better adherence to treatment, as well as better knowledge about diabetes and greater interest in performing, monitoring, and seeking health care. Therefore, this study aims to evaluate the effect of patient navigation on glycemic control, disease knowledge, adherence to self-care in people with type 1 diabetes mellitus.

**Methods:**

This is an intervention study using a single group pre-test post-test design, carried out in a tertiary public teaching hospital in Southern Brazil. Participants over 18 years of age and diagnosed with type 1 diabetes were included. In total, three teleconsultations and one face-to-face consultation were carried out, with three-month intervals, until completing one year of follow-up. The nurse navigator conducted diabetes education based on the guidelines of the Brazilian Diabetes Society and the Nursing Interventions Classification. The differences between glycated hemoglobin, adherence to self-care, and knowledge about initial and final diabetes were estimated to verify the effect of patient navigation by nurses, according to the tool applied in the first and last consultations. Interaction analyses between variables were also performed. Student’s t-test, Generalized Estimating Equations, Wilcoxon test, and McNemar test were used.

**Results:**

The final sample consisted of 152 participants, of which 85 (55.9%) were women, with a mean age of 45 ± 12 years, and diabetes duration of 23.6 ± 11.1 years. Nurse navigators conducted 812 teleconsultations and 158 face-to-face consultations. After the intervention, glycemic control improved in 37 (24.3%) participants (p < 0.001), and knowledge about diabetes also improved in 37 (24.3%) participants (p < 0.001). Adherence to self-care increased in 82 (53.9%) patients (p < 0.001). The analysis of the interaction between glycemic control and the results from the questionnaire of knowledge about diabetes showed an interaction effect (p = 0.005). However, we observed no interaction effect between glycemic control and the results from the questionnaire on adherence to self-care (p = 0.706).

**Conclusions:**

Our results showed improvement in glycemic control, adherence to self-care, and knowledge of diabetes in the study participants. In addition, they suggest that patient navigation performed by nurses is promising and feasible in improving care for patients with type 1 diabetes.

## Introduction

Patient navigation was introduced in the early 1990s to reduce unequal access to care for people with cancer [1]. The patient navigation model was then adapted to meet the complex health and social needs of other underserved populations [2, 3], especially those with chronic diseases, such as diabetes mellitus [4].

A study carried out on navigation of patients with type 2 diabetes (T2DM) showed that the group accompanied for guidance and education as a stimulus to adherence to treatment obtained positive results with reduced glycated hemoglobin (HbA1c) levels, better adherence to treatments, and lower body mass index after 12 weeks of intervention [5]. However, it is expected that frequent contact with patients with chronic diseases would have such an effect [6], thus, the absence of a control group is a major limitation of the study [5]. Brazilian studies have found that monitoring patients with diabetes remotely can have a beneficial effect on diabetes self-care. Remote monitoring can improve medication adherence, as by receiving regular reminders and notifications about taking medication, testing glucose levels, and other self-care tasks, patients are more likely to follow their treatment plan. Overall, remote monitoring was effective in helping patients with T2DM better manage their condition and improve their self-care [7, 8]. Other studies, with patients who received navigation for follow-up, found that patient navigation can lead to better adherence to treatment and better knowledge of the disease. Navigation for follow-up care typically involves providing patients with personalized support and guidance to help them navigate the healthcare system, manage their diabetes, and stay on track with their difficulties [5, 9, 10]. Moreover, studies also show that patients who received navigation for follow-up care have greater interest in performing, monitoring, and screening exams related to diabetes. Navigation programs often include regular check-in and follow-up appointments, which can keep patients engaged in their care and motivated to take an active role in managing their diabetes [5, 9, 10].

Patient navigation has been suggested as an innovative strategy to overcome barriers to the quality of care in the Brazilian Unified Health System (SUS) [11]. In Brazil, navigation for patients with chronic diseases is still under development, with no published studies on patient navigation for individuals with type 1 diabetes mellitus (T1DM). However, a navigation program for cancer patients has already been developed [11, 12], and a pilot project, which lasted one year and included 105 patients, showed an increased referral rate of patients before 60 days of diagnosis from 6 to 52% in cases of breast cancer [11, 13].

Patient navigation is an important differential and is usually performed by a nurse, but other professionals or non-clinical navigators can also do it [14, 15]. Besides acting as a care coordinator, the nurse can contribute to patient care by providing the necessary support to overcome the impact of treatment and overcome the main barriers that hinder access to health services and systems, such as cultural and language barriers, lack of knowledge, and difficulty in accessing health services [15].

The trajectory of patients with T1DM in health services is complex and may result in several consultations and exams [16]. These patients represent near 10% of those with diabetes mellitus, comprising 1.2 million people worldwide [17]. Good glycemic control is directly associated with lower incidence of chronic diabetic complications and mortality and is attained by frequent consultations with doctors, nurses, and nutritionists, proper insulin adjustments, and blood glucose self-monitoring, which requires high knowledge of the disease and adherence to treatment [18, 19]. Insulin dependence requires people with T1DM to be consistent in self-care to ensure stable blood glucose levels and reduce the risk of developing complications. Strict glycemic control, insulin supplementation, practice of routine physical activities, and the need to maintain regular eating habits, or deprivation at times, are the arduous and necessary practices of T1DM patients [20]. Thus, individuals with T1DM need to understand and develop skills to maintain this intense routine of care, and health professionals who serve this public need alternatives to help the patient on this journey.

Given the possible benefits of patient navigation for people with diabetes, this study aimed to evaluate the effect of patient navigation on glycemic control, disease knowledge, and adherence to self-care in people with T1DM.

## Methods

### Study design and setting

This is an intervention study using a single group pre-test post-test design, carried out in a tertiary public teaching hospital in Southern Brazil. The Transparent Reporting of Evaluations with Nonrandomized Designs (TREND) checklist was used to report the results [21].

Around 395,826 outpatient consultations are carried out yearly at this hospital, and in 2021, more than 67,000 teleconsultations [22] were carried out. The institution’s endocrinology outpatient clinic, which is the research field, consists of endocrinologists, nurses, social workers, and nutritionists. This clinic is a reference for the treatment of people with T1DM from different ages, and it treats patients from all regions of the state.

Before this study, the usual care for patients with T1DM in the researched institution was performed by nurses, endocrinologists, and nutritionists in individual appointments, some occurring simultaneously and others not. However, in 2019, many patients were left without multidisciplinary follow-up [23].

### Study population and sample size

The population consisted of patients diagnosed with T1DM, with regular follow-up at the institution’s endocrinology outpatient clinic. All patients with T1DM treated at the institution who had been treated at the endocrinology outpatient clinic in the previous two years were selected via a query request from electronic medical records with keywords in December 2020. To be included in the study, participants had to be older than 18 years old and diagnosed with T1DM. Exclusion criteria were having a record of another type of diabetes (T2DM, Maturity Onset Diabetes of the Young – MODY, Latent Autoimmune Diabetes in Adults – LADA, or uncertain type of diabetes), cognitive impairment, pregnancy, hearing loss, or chemical dependency.

The online version of the Power and Sample Size Health tool was used to estimate the power of the sample and was obtained by patients’ queries [24]. The power to test a minimum 0.5% difference in mean HbA1c differences between groups after *vs.* before the intervention was 86.5%. This value was obtained considering a 5% significance level, sample size equal to 152 pairs, and mean and standard deviation of differences of 0.4 and 1.6%, respectively, as obtained by Figueira et al. [25]. The power estimation to test if the proportion of participants who worsened and improved in adherence to self-care and knowledge about diabetes after the intervention was 100%.

### Instrument with validity and recruitment

**Fig. 1:**
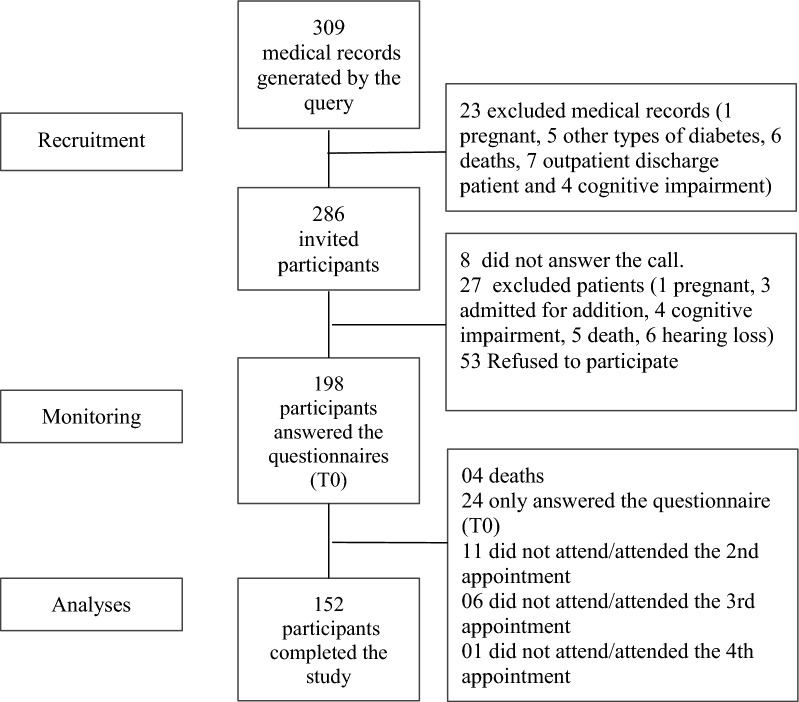
Study flowchart and sample constitution

Patients were selected from the electronic medical records query and totaled 309. After the first review, 286 met the inclusion criteria. The recruitment process took place by telephone due to the measures implemented to reduce the transmission of COVID-19. Of the 286 patients, 278 could be contacted. During the phone calls, the established criteria were applied again, and 27 people presented at least one of the exclusion criteria, which was not included in their medical record, and thus, they were excluded. Notably, 53 people refused to participate in the study. Thus, 198 people responded to the baseline questionnaires. Of these, 46 discontinued for the following reasons: 4 deaths and 42 dropouts during the intervention. Thus, 152 participants concluded the study (Fig. 1).

The study took place between January 2021 and April 2022. The invitation calls were made by the researchers during business hours, that is, from 8 a.m. to 6 p.m. Patients were asked about their interest in participating in the research and the availability of completing the questionnaires (Diabetes Knowledge Questionnaire and Self-Care Inventory Revised) on this first call or if they wished to reschedule. The phone calls were recorded, and the participants were asked to state if they agreed to participate in the research before the application of the questions. The questionnaires were repeated 30 days after the last consultation.

An online form was created to collect data on the variables studied to facilitate the completion of the participants’ answers, which included medical record number, telephone number, gender, age, schooling, time of diagnosis, active smoking, HbA1c, comorbidities (cardiovascular diseases, dyslipidemia, arterial hypertension, diabetes kidney disease, neuropathy, foot injuries, previous amputations, and psychiatric conditions), validated Diabetes Knowledge Questionnaire (DKN-A), and Self-Care Inventory Revised (SCI-R) translated and adapted to Brazilian Portuguese [26, 27]. Three questions about foot self-care were extracted from the Summary of Diabetes Self-Care Activities Questionnaire (SDSCA) [28], also translated and adapted to Brazilian Portuguese. The link to the questionnaire was sent to the participants who requested it, so that they could answer the questions, both before and after the intervention.

The DKN-A is a 15-item multiple-choice questionnaire on different aspects related to general knowledge of diabetes. Scale ranges from 0 to15 and each item is measured with a score of one (1) for correct answers and zero (0) for incorrect answers. Items one to 12 require a single correct answer. For items 13–15, some answers are correct, and all of them must be  answer correctly for participants to obtain a score of one. A score greater than eight indicates knowledge about diabetes [26]. Notably, in the results presentation, participants with scores from 0 to 8 were classified as “low knowledge” and above 9 as “satisfactory knowledge.”

The SCI-R has 14 items on a 5-point Likert scale (1 = never; 5 = always) that reflects how the participants followed the self-care recommendations during the last two months; higher scores indicate greater adherence, and the cut-off value to classify a patient as having a greater or lesser adherence score is 48 [27]. In this case, when presenting the results, participants with scores below 48 were referred to as having lesser adherence to self-care and scores above 49 as having greater adherence. And in the SDSCA, the response score is from 0 to 7 (0 = least desirable situation; 7 = most favorable) [28].

### Intervention

The first teleconsultation was scheduled for 30 days after the patients agreed to participate in the study and answered the questionnaire. In total, three teleconsultations and one face-to-face consultation were carried out, with three-month intervals, until completing one year of follow-up. The teleconsultations were carried out by nurses and a nursing student, at a time determined by the participant, in a specific room for this purpose. The face-to-face consultation was scheduled according to the patient’s availability to attend the hospital and after the release of the COVID-19 contingency measures. The teleconsultations and the face-to-face consultation lasted from 20 to 30 min and followed a script prepared by the researchers. Correspondence was sent to the patients who could not be contacted by phone to the address registered in the electronic medical record, so that they could come to the hospital to reschedule their appointments and update their records.

Patient navigation by the research nurses took place as follows: before the first teleconsultation, it was verified for how long the patient had not consulted with their doctor and nutritionist at the hospital and, if they had no appointment, they were instructed on how to reschedule one. In cases of difficulties with the hospital application or unfavorable financial conditions, the nurses scheduled the appointment. It was also verified in the electronic medical record that if the patient had recent HbA1c collection, and had they not performed the exam in the previous three months, a specific request was made to perform the exam. The same occurred at the end of the one-year follow-up. The nurse navigator provided a phone number for patients to contact in case of doubts and/or guidance.

During the teleconsultations and the face-to-face consultation, the nurse navigator applied diabetes education based on the guidelines of the Brazilian Diabetes Society (SBD) [29] and the Nursing Interventions Classification (NIC) [30], which were adapted according to the context and the participant’s conditions. The consultations considered the following subjects: first: health education, subcutaneous drug administration, prescribed drugs, and agreed realistic short and long-term goals; second: foot care and assessment and performance of the Semmes–Weinstein monofilament test (this consultation may vary according to the patient’s availability to attend face-to-face); third: improvement in coping, activities/exercise prescribed, improvement in willingness to learn, diet planning, and assistance to stop smoking when present; fourth: support for decision-making, guidelines regarding eye fundus examination, vaccination, and review on medication adherence, adherence to self-monitoring, and agreed goals. Furthermore, the nurse navigator sent notices electronically (e-mail/WhatsApp/message) regarding the dates of consultations and exams to be performed by the patients. In specific cases, additional phone contacts were made.

As part of the intervention, psychoeducation videos made available by the TelePSI project were sent to patients electronically (e-mail/WhatsApp) according to the preference reported by the participant [31]. The TelePSI project is an initiative of the Brazilian Ministry of Health in partnership with our Institution, which sought to offer psychological and psychiatric teleconsultation for the management of stress, anxiety, depression, and irritability in SUS professionals in the context of COVID-19. In total, 16 psychoeducation videos were produced and are freely available on the project’s website [31]. However, 11 psychoeducation videos were selected to comprise this intervention, considering its duration and the most relevant themes judged by the researchers.

The primary objective of the study was the difference in HbA1c levels (last one minus the first one). The secondary outcomes included improved knowledge of diabetes and adherence to self-care, measured by the DKN-A and SCI-R questionnaires and three foot self-care questions extracted from the SDSCA, which were applied before and after the intervention.

To establish adequacy or lack of it for glycemic control, individualized goals were used. Participants with a history of severe ischemic heart disease, frequent episodes of hypoglycemia, severe visual impairment, those who underwent hemodialysis or peritoneal dialysis, and underwent only two or fewer capillary blood glucose tests per day were considered for a flexible target (HbA1c ≤ 8.0%). For all other participants, strict glycemic control was considered adequate (HbA1c target ≤ 7.0%). Patients who were within the glycemic target were considered to have good control and the others to have inadequate glycemic control.

### Data analysis

Data analysis was performed using the Statistical Package for the Social Sciences (SPSS) version 22.0. Categorical variables were described by absolute number and percentile, and continuous variables were described by mean and standard deviation in case of normal distribution. Otherwise, data were described as median and interquartile range. Normality was defined by the Shapiro–Wilk test. The difference between initial and final HbA1c was estimated to evaluate the effect of patient navigation by nurses, as well as adherence to self-care and knowledge about diabetes according to the tool applied in the first and last consultation. Student’s *t*-test for samples and Generalized Estimating Equations with normal distribution were used. The Wilcoxon test was performed for paired samples with non-normal distribution, and the McNemar test was performed for categorical variables. The statistical significance level was 5%.

The post hoc exploratory analysis was performed by comparing patients who answered the questionnaires and did not complete the intervention with those who did. The two groups were compared for age, gender, duration of diabetes, and HbA1c levels.

### Ethical considerations

The study was approved by the ethics committee of the institution via Plataforma Brasil under CAAE No. 20380919800005327, considering the prerogatives announced in Resolution 466/2012 of the Brazilian National Health Council. The researchers followed the institution’s telephone call script for inviting participants to the research, which contained three options for the participant to choose to send an informed consent form (email, WhatsApp, or message), with the document being sent according to their preference. When handling the information, the researchers preserved the participants’ anonymity during the treatment and publication of the data.

## Results

Of the 198 participants who answered the questionnaires (T0), 106 (53.5%) were women, mean age 43 ± 12 years, diabetes duration 22 ± 10 years, mean HbA1c 8.6 ± 1.6%. Of those interviewed in the first telephone contact, 167 (84.3%) had no follow-up with the nurse, 63 (31.8%) were advised/helped to reschedule an appointment with the endocrinologist, 107 (54%) were advised/helped to reschedule an appointment with the nutritionist, and 44 (22.2%) were referred to the first appointment with the nutritionist. It is worth remembering that even patients who did not respond to the questionnaires were instructed to schedule an appointment with an endocrinologist, nutritionist, and diabetes educator nurse at the institution.

The final sample consisted of 152 participants, of which 85 (55.9%) were women, mean age 45 ± 12 years and diabetes duration 23.6 ± 11.1 years. When comparing patients who answered the questionnaires and did not complete the intervention with those who did, the post hoc analysis showed no difference between the number of male and female patients who dropped out and completed the study (p = 0.299). However, in patients who did not complete the survey, age (p = 0.036) and diabetes duration (p = 0.014) were lower, and HbA1c levels were higher (p = 0.048). Table 1 summarizes the other demographic and clinical characteristics of the patients who completed the study.

**Table 1:** Sociodemographic and clinical characteristics of study participants (n = 152)

Variables^a^	n = 152
Age (years)	45.3 ± 12.4
Woman	85 (55.9)
Marital status Married	95 (62.5)
Active tobacco use	17 (11.2)
Diabetes duration (years)	24 (14–31)
Professional occupation	
Employed	81 (53.3)
Unemployed	19 (12.5)
Retired	52 (34.2)
Schooling level	
Elementary school	35 (23.0)
High school	84 (55.3)
Higher education	33 (21.7)
Socioeconomic level	
Up to 1 minimum wage	36 (23.7)
From 1 to 3 minimum wages	81 (53.3)
Above 3 minimum wages	35 (23)
Injection device	
Pen	120 (78.9)
Syringe	32 (21.1)
Comorbidities^#^	
Systemic arterial hypertension	47 (30.9)
Psychiatric illnesses	47 (30.9)
Dyslipidemia	38 (25.0)
Diabetic retinopathy	85 (55.9)
Diabetic kidney disease	31 (20.4)
Sensory neuropathy	28 (18.4)
Foot injuries	13 (8.6)

^a^Continuous variables described by mean and standard deviation and categorical variables by absolute number and percentile. ^#^Comorbidities were considered present when listed in medical records. 1 minimum wage is approximately 275 USD

The nurse navigators conducted 812 teleconsultations and 158 face-to-face consultations. Two patients contacted the nurse navigator by phone when they were experiencing hypoglycemia and were instructed on how to proceed. The nurse navigators also helped a patient diagnosed with a tumor to obtain the correct referrals in a timely manner, as directed by the legislation in these cases. Four patients frequently sent messages via WhatsApp after consultation with the endocrinologist to clarify doubts regarding the prescription of insulin. A patient contacted the nurse referring to suicidal ideation. The nurse navigators managed the situation and the patient sought psychiatric care, as well as an appointment with the institution’s endocrinologist. Furthermore, several conversations were held via WhatsApp to confirm appointment dates, exams, how to proceed in cases of rescheduling, and to clarify doubts.

The analysis of the nurse navigator intervention regarding the primary outcome showed that 37 (24.3%) participants who had inadequate glycemic control before the intervention (T0) presented adequate glycemic control after the intervention (p = 0.001). Regarding knowledge about diabetes, 37 (24.3%) participants also had low knowledge at T0 and presented satisfactory knowledge post-intervention (p < 0.001). Considering adherence to self-care, the analysis showed that 82 (53.9%) patients who had lower adherence to self-care at T0 started to show greater adherence to self-care after the intervention (p < 0.001). Table 2 shows the primary and secondary outcomes.

**Table 2:** Intervention effect (patient navigation) on primary and secondary outcomes (n = 152)

Variables^a^	Pre-Intervention	Post-Intervention	p value
Primary outcomes			
HbA1c (%)	8.5 ± 1.5	8.3 ± 1.3	0.028†
Adequate glycemic control	30 (19.7)	54 (35.5)	0.001‡
Inadequate glycemic control	122 (80.3)	98 (64.5)	
Secondary outcomes			
Diabetes knowledge questionnaire	9.5 ± 2.5	12.4 ± 1.8	< 0.001†
Low knowledge	42 (27.6)	7 (4.6)	< 0.001‡
Satisfactory knowledge	110 (72.4)	145 (95.4)	
Revised self-care inventory	45.0 ± 7.4	54.1 ± 5.7	< 0.001†
Lesser self-care	101 (66.4)	20 (13.2)	< 0.001‡
Greater self-care	51 (33.6)	132 (86.8)	
Foot self-care			
Question 1	4 (1–7)	7 (5–7)	< 0.001 #
Question 2	2 (0–7)	7 (4–7)	< 0.001 #
Question 3	7 (5–7)	7 (7–7)	< 0.001 #

^a^Continuous variables described by mean and standard deviation; Categorical variables described by absolute number and percentage in case of normal distribution, and median and interquartile range in case of non-normal distribution. HbA1c: Glycated hemoglobin. Satisfactory knowledge was considered 9 or more correct answers; Greater self-care was considered a score greater than or equal to 49; Question 1: On how many of the previous seven days did you evaluate your feet?; Question 2: On how many of the previous seven days have you examined the inside of your shoes?; Question 3: On how many of the previous seven days did you dry your feet between the toes?; ^†^ Student’s *t-*test; ^‡^ McNemar test; ^#^ Wilcoxon test

The analysis of the interaction (patient navigation) in the primary outcome according to knowledge (DKN-A questionnaire) showed an interaction effect between glycemic control and the DKN-A questionnaire results (p = 0.005), since HbA1c levels decreased for participants with satisfactory knowledge (DKN-A ≥ 9), whereas patients with low knowledge (DKN-A ≤ 8) presented increased HbA1c levels. The analysis of the interaction (patient navigation) in the primary outcome according to adherence to self-care (SCI-R questionnaire) showed no interaction effect (p = 0.706), since the reduction in HbA1c levels among those who presented good self-care (SCI-R ≥ 49) and low self-care (SCI-R ≤ 48) was similar. The analysis of the interaction between glycemic control and gender showed an interaction effect (p = 0.019) due to improved HbA1c levels among women; however, men maintained the same HbA1c values. The analysis of the interaction between glycemic control and occupation showed a similar reduction in HbA1c levels among employed, unemployed, and retired participants. The reduction in HbA1c levels was similar among people with different schooling levels. Table 3 presents this information.

**Table 3:** Analysis of the intervention effect (patient navigation) on glycemic control according to knowledge about diabetes, adherence to self-care, gender, schooling level, and professional occupation (n = 152)

Variables^a^	HbA1c Pre-Intervention	HbA1c Post-Intervention	*p* value^†^
Diabetes knowledge questionnaire			
Low knowledge	8.4 ± 0.16 (n = 42)	9.1 ± 0.33 (n = 7)	0.028
Satisfactory knowledge	8.5 ± 0.14 (n = 110)	8.2 ± 0.10 (n = 145)	0.013
Self-care inventory revised			
Lesser self-care	8.4 ± 0.13 (n = 101)	8.1 ± 0.27 (n = 20)	0.219
Greater self-care	8.5 ± 0.19 (n = 51)	8.3 ± 0.11 (n = 132)	0.144
Gender			
Female (n = 85)	8.7 ± 0.18	8.3 ± 0.14	0.006
Male (n = 67)	8.2 ± 0.16	8.2 ± 0.15	0.823
Schooling			
Elementary school (n = 35)	8.9 ± 0.32	8.4 ± 0.27	
High school (n = 84)	8.4 ± 0.16	8.2 ± 0.13	0.059
Higher education (n = 33)	8.2 ± 0.21	8.1 ± 0.16	0.242
Socioeconomic level			
Up to 1 minimum wage	8.7 ± 0.30	8.4 ± 0.25	0.119
From 1 to 3 minimum wages	8.4 ± 0.17	8.3 ± 0.13	0.267
Above 3 minimum wages	8.4 ± 0.22	8.1 ± 0.19	0.131
Injection device			
Pen	8.6 ± 0.14	8.3 ± 0.11	0.005
Syringe	8.0 ± 0.25	8.1 ± 0.25	0.435
Occupation			
Active (n = 81)	8.4 ± 0.17	8.3 ± 0.14	0.256
Unemployed (n = 19)	8.5 ± 0.42	7.9 ± 0.22	0.105
Retired (n = 52)	8.5 ± 0.20	8.4 ± 0.18	0.228

^a^Categorical variables by absolute number and percentile. HbA1c: Glycated Hemoglobin; Satisfactory knowledge was considered 9 or more correct answers; Greater self-care was considered a score greater than or equal to 49; ^†^Post hoc test for comparing pre- and post-intervention intergroups. 1 minimum wage is approximately $275

The analyses were performed with two models adjusted for the HbA1c outcome, one of them is the diabetes knowledge questionnaire and the other is the revised self-care questionnaire. Both models were added to the variables: gender, schooling level, socioeconomic level, injection device, and occupation. With the adjustments, the results found, as well as the differences for both models, were similar to those reported in Table 3.

The analysis of the interaction of knowledge about diabetes according to foot care questions showed that both patients with either higher or lower knowledge improved their foot self-care (p < 0.001). The analyses showed that participants with low knowledge had a greater increase in foot care compared to patients with better knowledge at T0.

Regarding the videos sent, three (2%) patients could not watch them due to lack of internet access, one (0.7%) did not like them, 80 (52.6%) rated them as good, and 68 (44.7%) rated them as great.

## Discussion

This was an intervention study using a single group pre-test post-test to evaluate the effect of patient navigation on glycemic control, disease awareness, and adherence to self-care in people with T1DM. After 12 months of follow-up, we could show that the interventions of the nurse navigators were effective, since the glycemic control was better, as well as self-care adherence and diabetes knowledge in the patients who participated in the study.

Although the reduction in the HbA1c value was small (0.2% delta absolute reduction), almost 25% of the participants improved their glycemic control. However, unlike the study carried out in Alabama (United States), in which the patients reported their HbA1c value [5], the participants of the present study who did not have a recent record of this test in their medical records, performed the blood collection before and after the intervention. These data corroborate what was found in studies that evaluated glycemic control after patient navigation in individuals with diabetes [14, 32]. However, these studies included patients with T2DM and navigation was conducted by non-clinical patient navigators. The follow-up time of the patients was different from ours; one study was developed over nine months [32] and the other over two years [14]. The follow-up time in patient navigation can vary depending on the patient’s needs. Some navigation programs may involve regular visits with a navigator over an extended period, whereas others may have a more limited follow-up period.

Although we observed a greater reduction in HbA1c levels in the participants who were unemployed during data collection, no reduction was observed when compared with the other patients, perhaps due to the low number of people in this category. However, our analysis showed a reduction in HbA1c levels when verifying the variable. These data differ from the study carried out to identify the determinants of glycemic control among people with diabetes, which found worse glycemic control in unemployed people [33]. Regardless of living arrangement, whether working or unemployed, people who did not accept patient navigation monitoring had worse glycemic control [34], similar to our study.

Regarding knowledge about diabetes, although the average score of the DKN-A questionnaire was considered satisfactory, above nine correct answers, in the pre-intervention period, the participants increased this average after teleconsultations and consultations with the nurse navigator. The same occurred in a Brazilian study carried out with patients with T2DM who underwent educational interventions to assess knowledge about diabetes before and after the intervention [25] and in a study that evaluated the effect of nursing consultations in this population [35]. Low knowledge about diabetes can be associated with poor self-care practices [36]. Knowledge about diabetes is essential for proper care of the disease [37], however, although considered important, knowledge alone does not cause behavior changes.

Considering adherence to self-care, the participants went from low self-care to greater self-care, according to the SCI-R questionnaire, which shows greater self-care, with scores above 49. Studies have shown that the better diabetes self-care, the better adherence to treatment [37–39]. Adherence to self-care can be challenging for many individuals with diabetes, as it requires ongoing commitment and effort. Factors that can affect adherence to self-care include lack of knowledge about diabetes, difficulty understanding and following a treatment plan, lack of access to healthcare, and mental health issues such as depression and anxiety [38, 39]. Furthermore, patients with better diabetes self-care are more likely to accept referrals to education and navigation programs [34].

The study participants who had low knowledge about diabetes took less care of their feet before the intervention and, after it, their foot care improved. Foot care practices were similar in patients with T2DM [37], and different in a study that encompassed T1DM and T2DM [36]. In the cross-sectional study of 267 patients with diabetic foot ulcer, 53.9% showed good foot self-care [40]. An educational approach can increase foot self-care and foot care knowledge by up to 60% [41].

In our study, face-to-face consultations and teleconsultations were conducted by nurse navigators, although we did not perform an analysis of medical appointment and exam adherence, as we were still amid the COVID-19 pandemic and many patients were afraid to attend the hospital. Our study presents similar results to the cohort study with pre- and post-intervention periods with 392 patients, which showed that patient navigation was associated with improved glycemic control and better clinic engagement among people with diabetes; however, this study included participants with both types of diabetes [14]. Another study that evaluated a patient navigator via telehealth including 4066 patients with chronic illnesses showed that patients who received the navigator’s intervention doubled the odds ratio of attending their appointments and 11,387 USD in return over investment [42].

However, the development and implementation of a patient navigation program may require significant organizational changes. Hospital and clinic leaders must pay special attention to organizational culture, business processes, people and engagement, clinical quality, and safety factors considered critical for implementing successful strategic change initiatives in healthcare organizations [43].

This study has some limitations, such as its single-group design and the population belonging to only one tertiary care center. However, it is a reference in this type of care, with patients from all over the state. Notably, our sample power was 86.5% to show differences on the predetermined outcomes. Thus, the characteristics of the participants are representative of the population served by public hospitals of low- and middle-income countries, and the results should be explored considering cultural and economic aspects, which affect the management of the disease. Second, the lack of follow-up of some participants and the post hoc analyses of the comparisons between the patients who answered the questionnaires and did not complete the intervention with those who did show that these groups were different. Perhaps, younger people and those with shorter diabetes duration have more commitments (work and school) and do not have time to take care of their health. Another limitation is the use of three nurse navigators, and as a result, we could not determine to what extent the success of our intervention was influenced by personal characteristics. However, the tasks were standardized, and all nurses followed the same intervention script. Finally, the absence of a control group could have introduced bias when interpreting the results obtained.

## Conclusions

Our results showed improvement in glycemic control, adherence to self-care, and knowledge of diabetes after an intervention focused on patient navigation by nurses, which shows that this is a promising and feasible tool to improve T1DM care. Patients in low- and middle-income countries face multiple barriers to access essential services. However, improving care accessibility will not eliminate all the access problems for these patients, although patient navigation can help identify and address some barriers in order to improve care for T1DM patients. Providing individualized care using patient navigation can increase access to care, and improving patient health can lower the cost of care provided. Our study also showed that T1DM patient care can be extended by remote interventions led by nurses, with a focus on informative, educational, and psychosocial support to improve glycemic control, strengthen the capacity for self-care, and improve knowledge about the disease.

The intervention using patient navigation performed a systematic follow-up and health education, as well as a patient-centered approach and their needs. It should be noted the lack of a “gold standard” of health education for people with diabetes, so this study presents important contributions to clinical practice and shows the research needs in other health systems. We are convinced that having someone as a reference, receiving reminders, and the videos sent during the research stimulated patients to improve their ability to manage diabetes. Although patients are the transforming agent of their reality, easier access and the availability of nurse navigators contributed to the results of this study. To our knowledge, this is the first Brazilian study to show the effect of patient navigation in T1DM patients.

## Data Availability

The datasets used and/or analyzed during the current study can be made available by the corresponding author on reasonable request.

## Abbreviations

COVID-19: Coronavirus disease 2019
DKN-A: Diabetes Knowledge Questionnaire
HbA1c: Glycated Hemoglobin
LADA: Latent Autoimmune Diabetes in Adult
MODY: Maturity Onset Diabetes of the Young
SDSCA: Summary of Diabetes Self-Care Activities Questionnaire
SCI-R: Self-care Inventory Revised
SPSS: Statistical Package for Social Science
SUS: Brazilian Unified Health System
T1DM: Type 1 Diabetes Mellitus
T2DM: Type 2 Diabetes Mellitus
TREND: Transparent Reporting of Evaluations with Nonrandomized Designs
